# PKM2 is not required for pancreatic ductal adenocarcinoma

**DOI:** 10.1186/s40170-018-0188-1

**Published:** 2018-10-23

**Authors:** Alissandra L Hillis, Allison N Lau, Camille X Devoe, Talya L Dayton, Laura V Danai, Dolores Di Vizio, Matthew G Vander Heiden

**Affiliations:** 1 Koch Institute for Integrative Cancer Research and the Department of Biology, Massachusetts Institute of Technology, Cambridge, MA 02139 USA; 2 Department of Biochemistry and Molecular Biology, University of Massachusetts Amherst, Amherst, MA 01003 USA; 3 Departments of Surgery, Biomedical Sciences, and Pathology and Laboratory Medicine, Cedars-Sinai Medical Center, Los Angeles, CA USA; 4 Department of Medical Oncology, Dana-Farber Cancer Institute, Boston, MA 02115 USA

**Keywords:** PKM2, PDAC, Pyruvate kinase, Pancreatic cancer

## Abstract

**Background:**

While most cancer cells preferentially express the M2 isoform of the glycolytic enzyme pyruvate kinase (PKM2), PKM2 is dispensable for tumor development in several mouse cancer models. PKM2 is expressed in human pancreatic cancer, and there have been conflicting reports on the association of PKM2 expression and pancreatic cancer patient survival, but whether PKM2 is required for pancreatic cancer progression is unknown. To investigate the role of PKM2 in pancreatic cancer, we used a conditional allele to delete PKM2 in a mouse model of pancreatic ductal adenocarcinoma (PDAC).

**Results:**

PDAC tumors were initiated in *LSL-Kras*^*G12D/+*^*;Trp53*^flox/flox^*;Pdx-1-Cre* (KP^−/−^C) mice harboring a conditional *Pkm2* allele. Immunohistochemical analysis showed PKM2 expression in wild-type tumors and loss of PKM2 expression in tumors from *Pkm2* conditional mice. PKM2 deletion had no effect on overall survival or tumor size. Loss of PKM2 resulted in pyruvate kinase M1 (PKM1) expression, but did not affect the number of proliferating cells. These findings are consistent with results in other cancer models.

**Conclusions:**

PKM2 is not required for initiation or growth of PDAC tumors arising in the KP^−/−^C pancreatic cancer model. These findings suggest that, in this mouse PDAC model, PKM2 expression is not required for pancreatic tumor formation or progression.

**Electronic supplementary material:**

The online version of this article (10.1186/s40170-018-0188-1) contains supplementary material, which is available to authorized users.

## Introduction

Pyruvate kinase is an enzyme that catalyzes the final step of glycolysis to convert phosphoenolpyruvate and ADP to pyruvate and ATP. Pyruvate kinase has four isoforms encoded by two genes. The PKLR gene encodes PKL, which is expressed primarily in the liver, and PKR, which is expressed in erythrocytes [1]. The PKM gene encodes both the PKM1 and PKM2 isoforms, with isoform selected determined by alternate mRNA splicing to include either exon 9 or 10 [2, 3]. Both PKM isoforms catalyze the same reaction in glycolysis, but PKM1 has constitutively high catalytic activity, whereas the catalytic activity of PKM2 is allosterically regulated [2, 4]. As a glycolytic enzyme, PKM2 can exist in either a low activity state that promotes biosynthesis or in a high activity state that is similar to that of PKM1 and promotes oxidative glucose metabolism [2].

PKM2 is expressed in a variety of cancer types, and both metabolic and non-metabolic functions for PKM2 in cancer have been proposed [5–10]. However, accumulating evidence suggests that PKM2 is not required for the growth or progression of most tumors, and non-metabolic roles for PKM2 remain controversial [11–18]. For instance, deletion of PKM2 has been shown to promote tumor progression in breast cancer and medulloblastoma models [14, 15] and a recent study found that systemic PKM2 depletion (while maintaining PKM1 expression) promoted tumorigenesis [18]. However, other studies have found that PKM2 is dispensable for development of leukemia, liver cancer, colon cancer, lymphoma, lung cancer, and squamous cell carcinoma [16–18]. Thus, the role of PKM2 in cancer may depend on the genetic and/or environmental context.

Reports regarding the role of PKM2 in pancreatic cancer have also been conflicting. PKM2 has been reported to promote proliferation, migration, invasion, and angiogenesis and to decrease apoptosis in pancreatic cancer cell lines [19–21]. Other cell line studies have reported that PKM2 expression is important for gemcitabine resistance [22–26]. High PKM2 expression in human pancreatic tumors has been associated with larger tumor size, worse overall survival, and shorter recurrence-free survival of pancreatic cancer patients [20, 24, 27, 28]. However, one study reported that higher PKM2 expression was associated with longer overall survival of pancreatic cancer patients [29], and other analyses found that expression of PKM2 had no effect on overall survival in pancreatic cancer [30, 31].

Given the controversy surrounding the role of PKM2 in pancreatic tumors, we evaluated the importance of PKM2 in PDAC by crossing mice harboring a conditional *Pkm2* allele [14] to the *LSL-Kras*^*G12D/+*^*;Trp53*^flox/flox^*;Pdx-1-Cre* (KP^−/−^C) mouse PDAC model [32]. By comparing mice with and without PKM2 conditional alleles, we aimed to determine the requirement for PKM2 in pancreatic cancer.

## Results

**Fig. 1:**
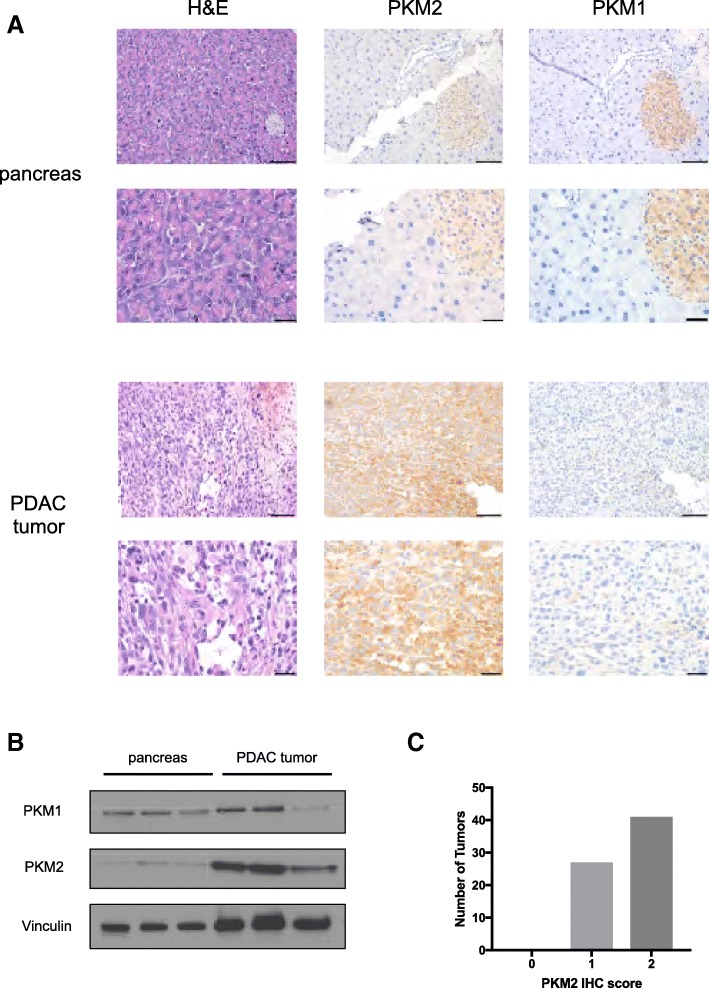
PKM2 is expressed in normal pancreas and in PDAC tumors. **a** Normal mouse pancreas and mouse PDAC tumor sections from end-stage *LSL-Kras*^*G12D/+*^*;Trp53*^flox/flox^*;Pdx-1-Cre* (KP^*−/−*^C) mice were stained with hematoxylin and eosin (H&E) and isoform-specific antibodies against PKM2 or PKM1 as shown. Scale bars represent 50 μm for all images at × 20 magnification (top) and 20 μm for all images at × 40 magnification (bottom). **b** Western blot of normal mouse pancreas and mouse PDAC tumor lysates (from KP^−/−^C mice) performed using isoform-specific antibodies against PKM1 and PKM2 as well as an antibody against vinculin as a control. **c** Distribution of PKM2 staining intensities from a tissue microarray containing sections from 68 human pancreatic adenocarcinoma tumors

To examine PKM2 isoform expression in the pancreas and in pancreatic tumors, we performed immunohistochemistry on normal mouse pancreatic tissue and mouse pancreatic tumor tissue sections using PKM2 or PKM1 isoform-specific antibodies [14]. PKM1 and PKM2 staining was prominent in the islets of normal mouse pancreas, whereas the rest of the pancreas exhibited minimal PKM1 or PKM2 expression (Fig. 1a), consistent with previously published reports [1]. Compared to the normal pancreas, end-stage PDAC tumors from *LSL-Kras*^*G12D/+*^*; Trp53*^flox/flox^*;Pdx-1-Cre* (KP^−/−^C) [32] (Fig. 1a) or *LSL-Kras*^*G12D/+*^*;LSL–Trp53*^R172H/+^*;Pdx-1-Cre* (KPC) [33] (Additional file 1: Figure S1A) mice showed increased staining for PKM2 and minimal PKM1 staining. This expression pattern was further verified by Western blot analysis of tissue lysates (Fig. 1b). To examine PKM2 expression in human pancreatic tumors, we performed PKM2 immunohistochemistry on 68 pancreatic tumor tissue samples using a tissue array. All of the samples analyzed stained positive for PKM2 expression (Fig. 1c).

**Fig. 2:**
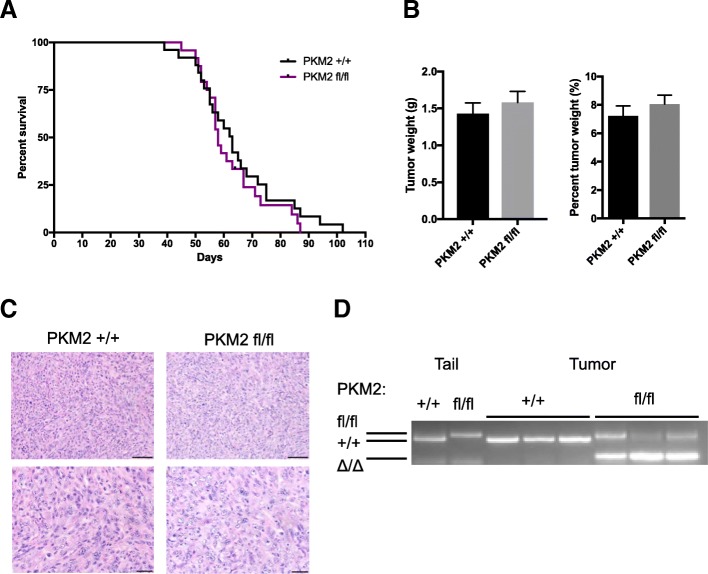
PKM2 deletion in PDAC tumors does not affect mouse survival, tumor weight, or tumor histology. **a** Kaplan-Meier curve showing survival of the *KP*^*−/−*^*C Pkm2*^+/+^ and *KP*^*−/−*^*C Pkm2*^flox/flox^ mouse cohorts. No statistically significant difference in survival was observed between the two cohorts (*n* = 24 *Pkm2*^flox/flox^ mice, 25 *Pkm2*^+/+^ mice per cohort, *p* = 0.3862, log-rank (Mantel-Cox) test). **b** The weight of tumors isolated from end-stage *KP*^*−/−*^*C Pkm2*^+/+^ and *KPC Pkm2*^flox/flox^ mice, and the percentage of the tumor weight relative to whole body weight is shown. **c** Hematoxylin and eosin (H&E) staining of PDAC tumors from *KP*^*−/−*^*C Pkm2*^+/+^ and *KP*^*−/−*^*C Pkm2*^flox/flox^ mice. **d** PCR genotyping of the PKM2 allele in tumors arising in *KP*^*−/−*^*C Pkm2*^+/+^ and *KP*^*−/−*^*C Pkm2*^flox/flox^ mice is shown. Analysis of tail DNA from *Pkm2*^*+/+*^ and *PKM2*^*flox/flox*^ mice is also shown as a control

To delete *Pkm2* in PDAC tumors, mice harboring a conditional *Pkm2* allele (*Pkm2*^*flox/flox*^) [14] were crossed to KP^−/−^C mice [32]. Overall survival was not significantly different between the KP^−/−^C;*Pkm2*^*+/+*^ mice and the KP^−/−^C;*Pkm2*^*flox/flox*^ mice (Fig. 2a), and there were no significant differences in weight of end-stage tumors in the KP^−/−^C;*Pkm2*^*+/+*^ mice compared to the KP^−/−^C;*Pkm2*^*flox/flox*^ mice (Fig. 2b). Histological analysis revealed no major differences between the KP^−/−^C;*Pkm2*^*+/+*^ tumors and KP^−/−^C;*Pkm2*^*flox/flox*^ tumors (Fig. 2c). To verify that *Pkm2* was deleted in tumors from KP^−/−^C;*Pkm2*^*flox/flox*^ mice, we analyzed genomic DNA from KP^−/−^C;*Pkm2*^*+/+*^ and KP^−/-^C;*Pkm2*^*flox/flox*^ pancreatic tumors and confirmed that PKM2 deletion had occurred in tumors arising in KP^−/−^C;*Pkm2*^*flox/flox*^ mice (Fig. 2d).

**Fig. 3:**
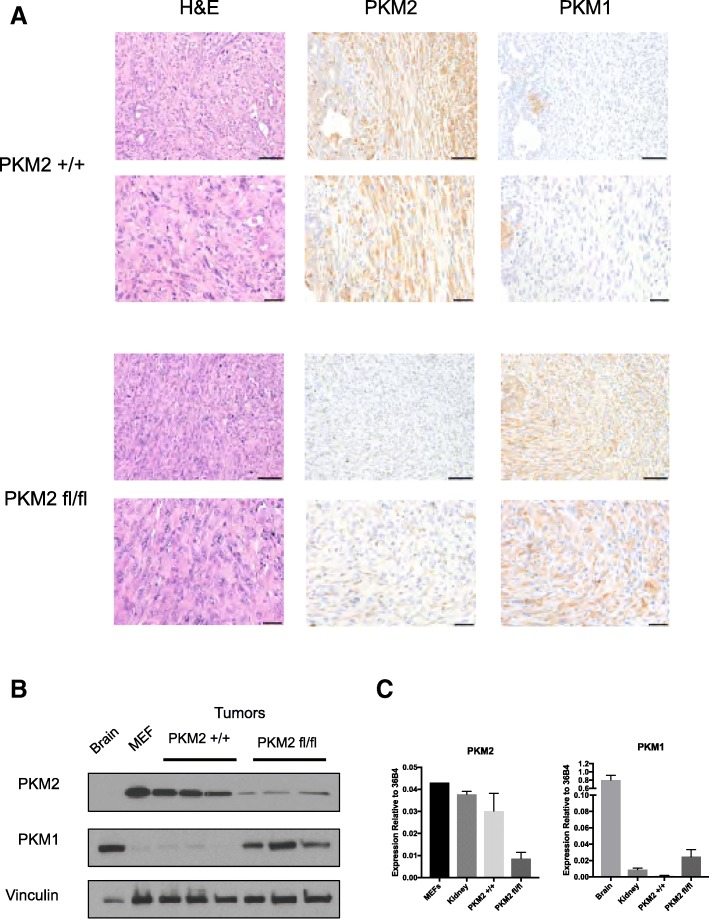
PKM2 deletion in PDAC tumors results in increased PKM1 expression relative to wild-type tumors. **a** Tissue sections from tumors arising in end-stage *KP*^*−/−*^*C Pkm2*^+/+^ and *KP*^*−/−*^*C Pkm2*^flox/flox^ mice were stained with hematoxylin and eosin (H&E) and isoform-specific antibodies against PKM2 or PKM1 as shown. Scale bars represent 50 μm for all images at × 20 magnification (top) and 20 μm for all images at × 40 magnification (bottom). **b** Western blot of tumor lysates from *KP*^*−/−*^*C Pkm2*^+/+^ and *KP*^*−/−*^*C Pkm2*^flox/flox^ mice was performed using isoform-specific antibodies against PKM2 or PKM1 as well as an antibody against vinculin as a control. **c** PKM2 and PKM1 expression was assessed by qPCR in tumors arising in *KP*^*−/−*^*C Pkm2*^+/+^ and *KP*^*−/−*^*C Pkm2*^flox/flox^ mice

We also analyzed pyruvate kinase expression in tumors isolated from KP^−/−^C;*Pkm2*^*+/+*^ and KP^−/−^C;*Pkm2*^*flox/flox*^ mice using immunohistochemistry with pyruvate kinase isoform-specific antibodies. As expected, the analysis of pancreatic tumors showed that KP^−/−^C;*Pkm2*^*+/+*^ tumors expressed almost exclusively PKM2 (Fig. 3a), whereas consistent with our genomic DNA analysis, tumors from KP^−/−^C;*Pkm2*^*flox/flox*^ mice showed no evidence of PKM2 expression in most cells (Fig. 3a). Tumors from KP^−/−^C;*Pkm2*^*flox/flox*^ animals showed increased PKM1 expression compared to KP^−/−^C;*Pkm2*^*+/+*^ wild-type tumors (Fig. 3a). To quantify pyruvate kinase expression in these tumors, we performed Western blot and qPCR analysis. Western blotting of KP^−/−^C;*Pkm2*^*+/+*^ tumor lysates showed that these tumors almost exclusively expressed PKM2 (Fig. 3b); qPCR analysis of KP^−/−^C;*Pkm2*^*+/+*^ tumor RNA also showed PKM2 expression and very little PKM1 expression (Fig. 3c). Western blotting of KP^−/−^C;*Pkm2*^*flox/flox*^ tumor lysates showed low PKM2 expression and high PKM1 expression (Fig. 3b). Similarly, qPCR analysis of KP^−/−^C;*Pkm2*^*flox/flox*^ tumor RNA showed increased PKM1 expression and decreased PKM2 expression, although PKM1 mRNA expression was much lower than that observed in the brain, a tissue that normally expresses PKM1 [1] (Fig. 3c). To investigate whether PKLR expression was induced by PKM2 loss, we also performed Western blot and qPCR analysis for PKLR. Neither KP^−/−^C;*Pkm2*^*+/+*^ nor KP^−/−^C;*Pkm2*^*flox/flox*^ tumor lysates exhibited evidence for PKLR expression at the RNA or protein level (Additional file 1: Figure S2A, B). qPCR analysis was also performed for PKM2 skip, a PKM isoform that excludes exons 9 and 10, which has been reported to be expressed in *PKM2*^*flox/flox*^ mice in other models [14]. KP^−/−^C;*Pkm2*^*+/+*^ tumors did not express PKM2 skip, while KP^−/−^C;*Pkm2*^*flox/flox*^ tumors expressed PKM2 skip message at detectable levels (Additional file 1: Figure S2C).

**Fig. 4:**
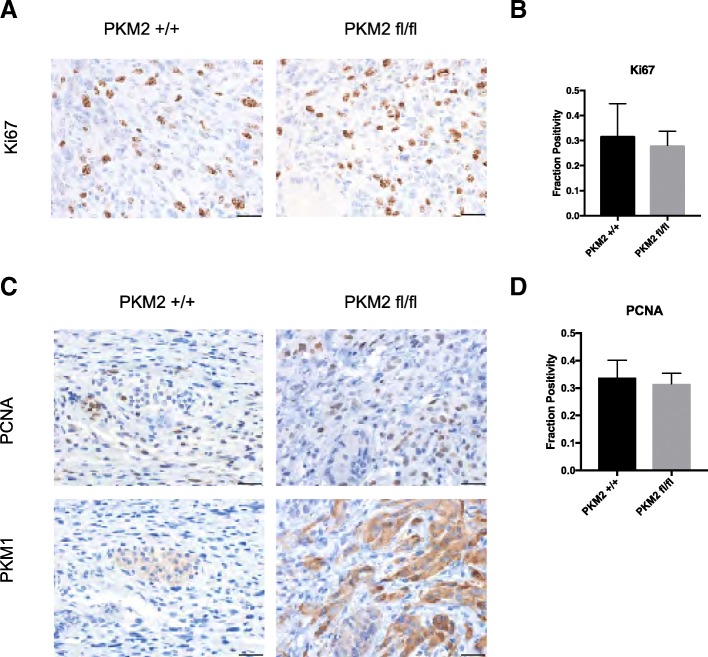
PKM2 deletion does not alter PDAC tumor cell proliferation. **a** Tumor tissue sections from end-stage *KP*^*−/−*^*C Pkm2*^+/+^ and *KP*^*−/−*^*C Pkm2*^flox/flox^ mice were stained with an antibody against Ki67 as shown. Scale bars represent 50 μm for all images at × 20 magnification (top) and 20 μm for all images at × 40 magnification (bottom). **b** Fraction of nuclei positive for Ki67 was assessed as shown. No significant difference in Ki67 positivity was observed in tumors arising in *KP*^*−/−*^*C Pkm2*^+/+^ and *KP*^*−/−*^*C Pkm2*^flox/flox^ mice. **c** Tumor tissue sections from *KP*^*−/−*^*C Pkm2*^+/+^ and *KP*^*−/−*^*C Pkm2*^flox/flox^ mice were stained with isoform-specific antibodies against PKM1 and against PCNA as shown. Scale bars represent 50 μm for all images at × 20 magnification (top) and 20 μm for all images at × 40 magnification (bottom). **d** Fraction of nuclei positive for PCNA was assessed as shown. No significant difference in PCNA positivity was observed in tumors arising in *KP*^*−/−*^*C Pkm2*^+/+^ and *KP*^*−/−*^*C Pkm2*^flox/flox^ mice

To compare cell proliferation in KP^−/−^C;*Pkm2*^*+/+*^ and KP^−/−^C;*Pkm2*^*flox/flox*^ tumors, we performed immunohistochemical staining with antibodies against the proliferative markers Ki67 or PCNA (Fig. 4a–d). There was no statistically significant difference in Ki67 or PCNA staining between KP^−/−^C;*Pkm2*^*+/+*^ and KP^−/−^C;*Pkm2*^*flox/flox*^ tumors (Fig. 4b, d). To determine if the proliferating cells expressed pyruvate kinase, we stained serial sections for PCNA or PKM1 (Fig. 4c). KP^−/−^C;*Pkm2*^*+/+*^ tumors had low PKM1 expression, and areas with high PKM1 expression did not correlate with areas of PCNA expression. KP^−/−^C;*Pkm2*^*flox/flox*^ tumors had higher PKM1 expression, and these tumors had many regions with only either PCNA or PKM1 expression and some regions with both PKM1 and PCNA expression. Consistent with previous reports, proliferating cells in *Pkm2*^*flox/flox*^ tumors may downregulate expression of pyruvate kinase to enable tumor growth.

## Discussion

These results argue that PKM2 expression has no significant effect on tumor initiation or progression in a mouse pancreatic cancer model driven by mutant *Kras* and deletion of both copies of *Trp53*. Although PKM2 is highly expressed in both mouse and human PDAC tumors, deletion of PKM2 had no effect on tumor size or survival of PDAC tumor-bearing mice. Our findings are consistent with meta-analyses of published datasets examining PKM2 expression and survival of human pancreatic cancer patients, which found that expression of PKM2 had no effect on overall survival [30, 31].

Our findings that PKM2 expression is not required for pancreatic tumor growth are consistent with findings from other cancer models showing that PKM2 is similarly not required for tumor development. This includes mouse models of colon cancer [12], breast cancer [14], medulloblastoma [15], leukemia [16], hepatocellular carcinoma [17, 18], lymphoma [18], lung cancer [18], and squamous cell carcinoma [18]. While deletion of PKM2 accelerated breast cancer and medulloblastoma progression and promoted hepatocellular carcinoma [14, 15, 17], loss of PKM2 slowed leukemia progression [16] and had no effect on progression of colon cancer [12]. A recent report using PKM1 and PKM2 knock-in mouse models showed that mice that exclusively expressed PKM1 had higher tumor burden than PKM2-expressing mice in a lung adenocarcinoma model [18]. Additionally, these whole-body PKM1-expressing mice had higher tumor incidence than PKM2-expressing mice in a carcinogen-induced mouse model [18]. We find here that deletion of PKM2 does not impair PDAC tumorigenesis or affect mouse survival or tumor size, suggesting that whether loss of PKM2 accelerates or slows cancer depends on the tissue and environmental context.

PKM2 expression was relatively high in wild-type PDAC tumors, whereas PKM1 expression was low in these tumors. PKM2 deletion in tumors resulted in an isoform switch from PKM2 to PKM1 expression. This result is consistent with findings observed in colon cancer [12], breast cancer [14], and leukemia [16] and suggests that PKM2 is dispensable in a variety of tumor types. PKM1 expression has been associated with decreased proliferative capacity in some systems [5, 6, 14] although a recent study reported that PKM1 expression can also be associated with increased proliferation in tumors [18]. Although we observed higher PKM1 expression in PKM2 deleted tumors compared to wild-type tumors, this did not affect the percentage of proliferative cells in the tumor or overall animal survival.

Differences between our results and previously published reports may be attributed to the possibility that PKM2 was not efficiently deleted from all of the PDAC tumor cells, despite the fact that we observed a robust decrease in PKM2 expression in these tumors. Additionally, it is likely that tumor stromal cells, which make up a significant percentage of PDAC tumors [34], retained PKM2 expression. Furthermore, it will be interesting to investigate PKM2 loss in other mouse models of pancreatic cancer driven by *Kras* with wildtype, heterozygous, or point mutant *Trp53* [32, 33]. Different metabolic requirements may exist in these tumors that arise more gradually, such as a differential dependence on autophagy [35]. Nonetheless, the loss of PKM2 expression in most tumor cells in a mouse PDAC model driven by mutant *Kras* and early homozygous loss of *Trp53* supports the notion that PKM2 is not generally required for PDAC initiation and progression. Although PKM2 is highly expressed in mouse and human PDAC, our findings support a model where PKM2 is dispensable for the growth of these cancers.

## Conclusions

Our study found that PKM2 is not required for KP^−/−^C pancreatic tumor growth or progression. Although PKM2 was found to be highly expressed in mouse and human PDAC tumors, loss of PKM2 in KP^−/−^C mice did not significantly affect overall survival, tumor size, or proliferative index. Loss of PKM2 in PDAC tumors resulted in higher PKM1 expression, but did not change cell proliferation, further arguing that PKM2 is not required for pancreatic tumor growth.

## Methods

### Mouse model and survival curve

*Pkm2*^*flox/flox*^ mice [14] were bred to the *LSL-Kras*^*G12D/+*^*;Trp53*^flox/flox^*;Pdx-1-Cre* (KP^−/−^C) mice [32] to generate animals of relevant genotypes. Male *Pkm2*^*flox/flox*^ and *Pkm2*^*+/+*^ mice were used for Kaplan-Meier survival curve and tumor weight analysis.

### Western blot

Western blots were performed using primary antibodies against PKM2 (Cell Signaling Technologies #4053, 1:1000 dilution), PKM1 (Cell Signaling Technologies #7067, 1:1000 dilution), PKLR (Santa Cruz #sc-133222, 1:1000 dilution), or vinculin (Abcam ab18058, 1:1000 dilution).

### Immunohistochemistry

Sections from formalin-fixed paraffin-embedded mouse tissue were stained with hematoxylin and eosin or with antibodies against PKM2 (Cell Signaling Technologies #4053, 1:800 dilution), PKM1 (Cell Signaling Technologies #7067, 1:500 dilution), Ki67 (BD Biosciences #550609, 1:40 dilution), or PCNA (Cell Signaling Technologies #2586, 1:4000 dilution). Percent positivity of Ki67 or PCNA staining was calculated using a positive pixel count algorithm in ImageScope software (Leica Biosystems). A human pancreatic cancer tissue microarray (Biomax PA961a) was stained for PKM2 positivity and scored independently by a pathologist (D.D.V.).

### qPCR

qPCR reactions were performed using SYBR Green Master Mix (Sigma) and primers for PKM1 (Forward: 5′- GTC TGG AGA AAC AGC CAA GG -3′, Reverse: 5′- TCT TCA AAC AGC AGA CGG TG -3′), PKM2 (Forward: 5′-GTC TGG AGA AAC AGC CAA GG -3′, Reverse: 5′- CGG AGT TCC TCG AAT AGC TG -3′), PKLR (Forward: 5′- AAG GGT CCC GAG ATA CGC A -3′, Reverse: 5′- CTG CAA CGA CCT GGG TGA TA -3′), and PKM-skip (Forward: 5′- ATG CTG GAG AGC ATG ATC AAG AAG CCA -3′ Reverse: 5′- CAA CAT CCA TGG CCA AGT T -3′) using 36B4 (Forward: 5′- TCC AGG CTT TGG GCA TCA -3′, Reverse: 5′- CTT TAT CAG CTG CAC ATC ACT CAG A -3′) as a control.

## Additional file

Additional file 1: **Figure S1.** PKM2 is expressed in KPC tumors. A) Sections from tumors arising in *LSL-Kras*^*G12D/+*^*;LSL–Trp53*^R172H/+^*;Pdx-1-Cre* (KPC) mice were stained with Hematoxylin & Eosin (H&E) and isoform-specific antibodies against PKM2 or PKM1 as shown. Scale bars represent 50 μm for all images at × 20 magnification (top) and 20 μm for all images at × 40 magnification (bottom). **Figure S2.** PKM2 deletion leads to expression of PKM skip, but does not induce PKLR expression. A) Western blot analysis of lysates from tumors arising in *KP*^*−/−*^*C Pkm2*^+/+^ and *KP*^*−/−*^*C Pkm2*^flox/flox^ mice performed using an isoform-specific antibody against PKLR and an antibody against vinculin as a control. B) PKLR expression was measured by qPCR of mRNA isolated from tumors arising in *KP*^*−/−*^*C Pkm2*^+/+^ and *KP*^*−/−*^*C Pkm2*^flox/flox^ mice. C) PKM-skip expression was measured by qPCR of mRNA isolated from tumors arising in *KP*^*−/−*^*C Pkm2*^+/+^ and *KP*^*−/−*^*C Pkm2*^flox/flox^ mice. (PDF 165 kb)

## Abbreviations

PDAC: Pancreatic ductal adenocarcinoma
PKLR: Pyruvate kinase, L/R isoform
PKM1: Pyruvate kinase, M1 isoform
PKM2: Pyruvate kinase, M2 isoform
