# Supplementary figures and images for: PKM2 is not required for pancreatic ductal adenocarcinoma
Source: Cancer Metab. 2018 Oct 23;6:17. doi: 10.1186/s40170-018-0188-1 (PMC6198443; doi:10.1186/s40170-018-0188-1)

A

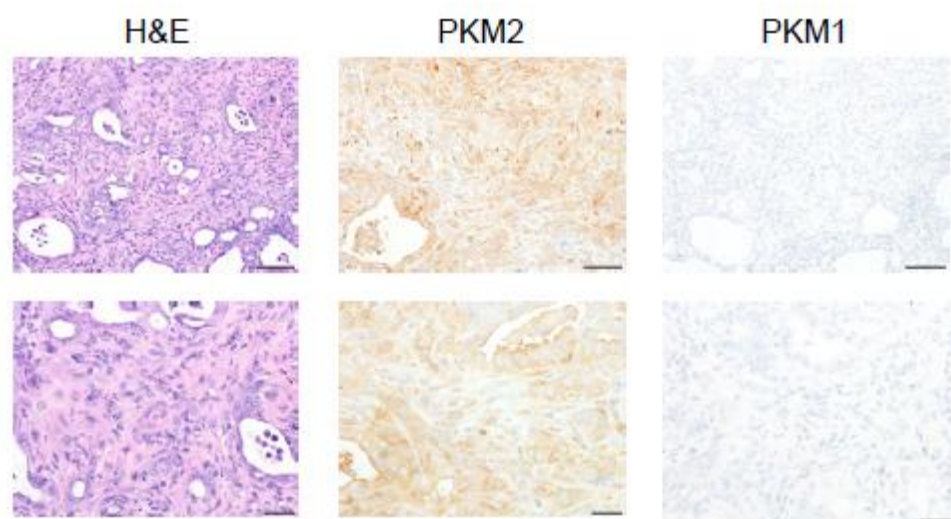

Figure S1

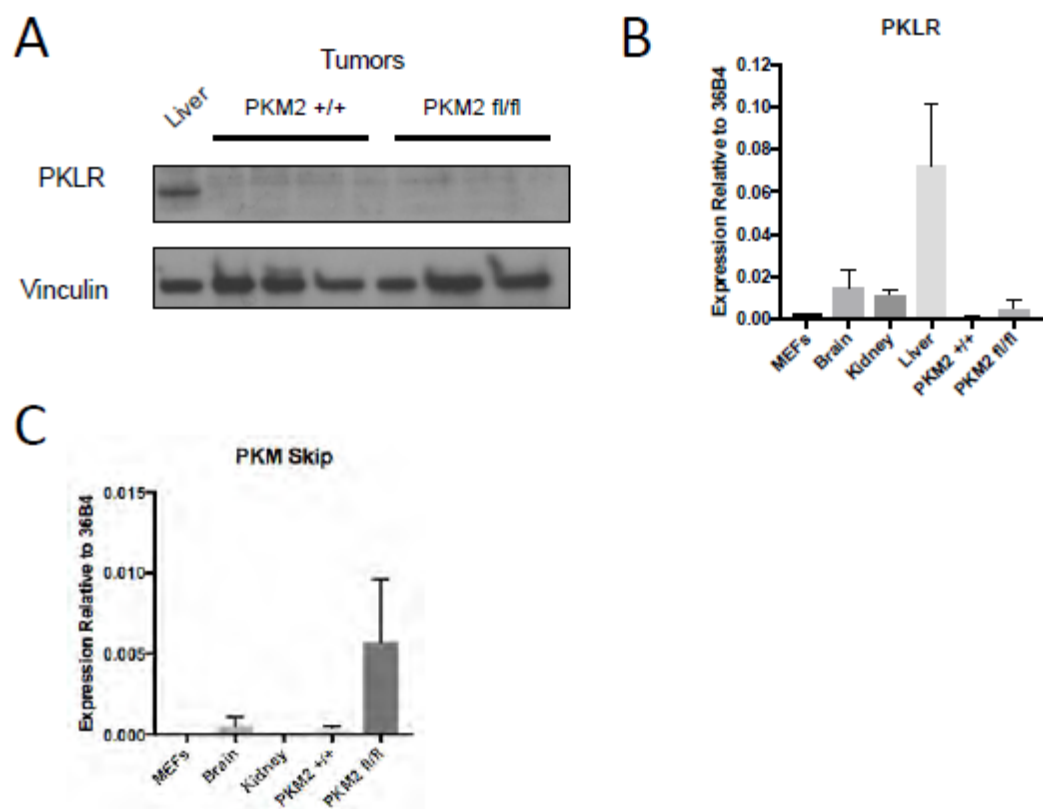

Figure S2

Supplement: Supplementary file 1 — Figure S1. PKM2 is expressed in KPC tumors. A) Sections from tumors arising in LSL-KrasG12D/+;LSL–Trp53R172H/+;Pdx-1-Cre (KPC) mice were stained with Hematoxylin & Eosin (H&E) and isoform-specific antibodies against PKM2 or PKM1 as shown. Scale bars represent 50 μm for all images at × 20 magnification (top) and 20 μm for all images at × 40 magnification (bottom). Figure S2. PKM2 deletion leads to expression of PKM skip, but does not induce PKLR expression. A) Western blot analysis of lysates from tumors arising in KP−/−C Pkm2+/+ and KP−/−C Pkm2flox/flox mice performed using an isoform-specific antibody against PKLR and an antibody against vinculin as a control. B) PKLR expression was measured by qPCR of mRNA isolated from tumors arising in KP−/−C Pkm2+/+ and KP−/−C Pkm2flox/flox mice. C) PKM-skip expression was measured by qPCR of mRNA isolated from tumors arising in KP−/−C Pkm2+/+ and KP−/−C Pkm2flox/flox mice. (PDF 165 kb) [file 40170_2018_188_MOESM1_ESM.pdf]
